# Salvage Chemoradiotherapy for Loco-Regional Recurrence of Esophageal Squamous Cell Carcinoma After Esophagectomy

**DOI:** 10.3390/jcm14051540

**Published:** 2025-02-25

**Authors:** Atsuto Katano, Tomoki Kiritoshi, Subaru Sawayanagi, Hideomi Yamashita

**Affiliations:** 1 Department of Radiology, The University of Tokyo Hospital, 7-3-1 Hongo, Bunkyo-ku, Tokyo 113-0033, Japan; 2 Department of Radiation Oncology, Nerima Hikarigaoka Hospital, 2-11-1 Hikarigaoka, Nerima-Ku, Tokyo 179-0072, Japan

**Keywords:** salvage chemoradiotherapy, esophageal cancer, loco-regional recurrence, prognostic factors, overall survival, progression-free survival

## Abstract

**Background/Objectives**: Loco-regional recurrence (LRR) of esophageal cancer following esophagectomy presents a significant therapeutic problem. This study aimed to evaluate the effectiveness of salvage concurrent chemoradiotherapy (CCRT) and to identify the prognostic factors influencing the survival outcomes in patients with an LRR of esophageal cancer. **Methods:** This retrospective study included 68 patients who underwent salvage CCRT for an LRR of esophageal squamous cell carcinoma between April 2008 and June 2024. Patients were treated with either 50.4 Gy in 28 fractions or 60 Gy in 30 fractions, along with concurrent fluoropyrimidine- and platinum-based chemotherapy. Prognostic factors were identified using univariate and multivariate Cox proportional hazards models. **Results:** The median overall survival (OS) was 30.1 months (95% confidence interval [CI]: 21.5–110.7 months), with a 2-year OS rate of 57.4%. The median progression-free survival (PFS) was 8.9 months (95% CI: 6.3–17.7 months). In the multivariate analysis, the significant prognostic factors for OS included the interval to recurrence (>1 year vs. ≤1 year, hazard ratio [HR] = 2.307, *p* = 0.024) and radiotherapy dose (60 Gy vs. 50.4 Gy, HR = 2.414, *p* = 0.040). For PFS, the interval to recurrence and radiotherapy dose remained significant predictors (*p* < 0.05). The 2-year OS rate was higher in the 60 Gy arm (62.7% vs. 42.0%, *p* = 0.285) and in patients with recurrence occurring >1 year after surgery (73.4% vs. 29.9%, *p* = 0.0054). The local control rate at 2 years was 71.9%, with better outcomes observed in the 60 Gy arm (93.5% vs. 76.5%, *p* = 0.0651). **Conclusions**: Salvage CCRT is a viable treatment option for LRR of esophageal cancer, achieving favorable survival outcomes, particularly in patients with late recurrence (>1 year) and in those receiving higher radiotherapy doses.

## 1. Introduction

A loco-regional recurrence (LRR) of esophageal cancer after esophagectomy presents a significant therapeutic challenge. Despite advances in surgical techniques and perioperative care, LRR rates remain high at approximately 40% [1,2]. The prognosis after recurrence is typically poor, with reported median survival durations ranging from 3 to 13 months [3,4,5]. This emphasizes the critical need for an effective salvage treatment to improve the outcomes of this aggressive disease [6].

Systemic chemotherapy is the standard approach for recurrent esophageal cancer and traditionally relies on fluoropyrimidine plus platinum-based regimens [7,8]. Recently, immune checkpoint inhibitor-based regimens have been demonstrated to improve overall survival in patients with recurrent esophageal squamous cell carcinoma. The KEYNOTE-590 trial reported a median overall survival (OS) of 12.4 months (95% confidence interval [CI]: 10.5–14.0) in the pembrolizumab plus chemotherapy group, compared to 9.8 months (95% CI: 8.8–10.8) in the chemotherapy only group (*p* < 0.0001) [9]. Similarly, the CheckMate 648 trial showed that both first-line nivolumab plus chemotherapy and nivolumab plus ipilimumab significantly prolonged overall survival compared to chemotherapy alone [10].

Evidence supporting the use of salvage concurrent chemoradiotherapy (CCRT) for LRR of esophageal cancer is also expanding. This study aims to evaluate the effectiveness of salvage CCRT and to identify prognostic factors influencing survival outcomes in patients with an LRR of esophageal cancer.

## 2. Materials and Methods

This study retrospectively analyzed patients with an LRR of esophageal cancer who underwent salvage CCRT following esophagectomy between April 2008 and June 2024. Locoregional recurrence was diagnosed based on radiological findings, not on clinical symptoms alone, and biopsy was not mandatory for all cases. Eligible patients met the following inclusion criteria: pathologically confirmed squamous cell carcinoma, recurrence confined to locoregional sites without distant metastasis, and salvage CCRT administered as first-line salvage therapy, while those with polymetastatic recurrent disease who were initially treated with chemotherapy and subsequently underwent CCRT for residual lesions were excluded. Additionally, patients with a history of thoracic radiotherapy or with incomplete clinical records that hindered the evaluation of staging, treatment details, or outcomes were excluded.

This study was conducted in accordance with the Declaration of Helsinki and approved by the Research Ethics Committee of the Graduate School of Medicine and Faculty of Medicine, University of Tokyo (approval number: 3372-8). Recurrence locations were categorized as anastomotic, cervical lymph nodes, thoracic lymph nodes, and abdominal lymph nodes. The time interval from esophagectomy to recurrence was documented in all cases. Radiotherapy was delivered using either three-dimensional conformal radiotherapy (3D-CRT) or intensity-modulated radiotherapy (IMRT), depending on the patient and disease characteristics. The clinical target volume included a detectable anastomotic site or a regional lymph node with recurrence. Prophylactic nodal irradiation was not administered to patients with clinically uninvolved lymph nodes.

Two radiotherapy dose regimens were utilized: 50.4 Gy in 28 fractions and 60 Gy in 30 fractions, which were selected based on the clinician’s decision at the time of treatment planning. Chemotherapy regimens were determined through multidisciplinary conferences and involved a combination of fluoropyrimidine-based antimetabolites and platinum-based agents. Nedaplatin (NDP), an analog of cisplatin, was developed to reduce cisplatin-induced toxicities such as nephrotoxicity and gastrointestinal toxicity [11]. This cisplatin (CDDP) derivative was designed to provide similar efficacy while minimizing side effects, and has been used in recent clinical trials [12,13]. S-1 is an oral fluoropyrimidine anticancer drug composed of tegafur, gimestat, and oteracil potassium in a 1:0.4:1 molar ratio [14]. S-1 has been widely studied in recent clinical trials, particularly in East Asia [15,16,17]. The CCRT regimen consisted of two cycles of chemotherapy administered concurrently with radiotherapy, followed by two additional cycles after radiotherapy, for a total of four cycles. Each NDP + S-1 cycle included NDP at 80 mg/m^2^, administered intravenously on day 1, and S-1 at 80 mg/m^2^/day, taken orally on days 1–14. For the NDP + 5-FU regimen, 5-FU at 800 mg/m^2^ was administered intravenously on days 1–5 instead of S-1. For the NDP regimen, fluoropyrimidine-based chemotherapy was omitted. For the CDDP + 5-FU regimen, CDDP at 70 mg/m^2^ was administered intravenously on day 1, while 5-FU at 700 mg/m^2^ was administered intravenously on days 1–5. For elderly patients or those with impaired renal function, dose reduction up to 80% was permitted.

Data analysis was conducted using the R statistical software version 4.4.2 (R Foundation for Statistical Computing, Vienna, Austria). Kaplan–Meier survival analysis was used to measure OS and progression-free survival (PFS). OS was defined as the time from the initiation of salvage CCRT to death from any cause, while PFS was defined as the time from the start of salvage CCRT to radiologically confirmed disease progression or death. Survival rates at one and two years were calculated with 95% confidence intervals (CIs). Prognostic factors for OS and PFS were evaluated using univariate and multivariate Cox proportional hazards models. Variables assessed included age, gender, ECOG performance status, radiotherapy dose, chemotherapy regimen, recurrence pattern, and interval from surgery to recurrence. Statistical significance was set at *p* < 0.05.

## 3. Results

A total of 68 patients with recurrent esophageal cancer who underwent salvage CCRT were included in this study. Patient characteristics are summarized in Table 1. The median time to recurrence was 12.8 months (range: 3.2–227.3 months). The median age of the cohort was 68 years (range: 41–83 years). The majority of the patients were male (56 patients, 82%), and most had a good performance status (ECOG PS 0), also accounting for 82% of the cohort. Thoracic lymph nodes were the most common sites of recurrence (29 patients, 43%), followed by anastomotic recurrence (13 patients, 19%), cervical lymph node recurrence (13 patients, 19%), and abdominal lymph node recurrence (13 patients, 19%). Regarding radiotherapy dose and fractionation, 28 patients (41%) received 50.4 Gy in 28 fractions, whereas 40 patients (59%) received 60 Gy in 30 fractions.

**Figure 1:**
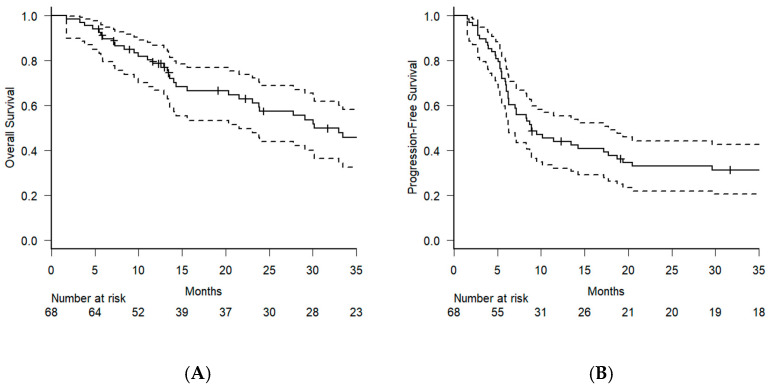
The solid lines indicate the Kaplan–Meier curves for overall survival (**A**) and progression-free survival (**B**). Dashed lines represent 95% confidence intervals. The number of people at risk at various time points is displayed below each graph.

The median follow-up period was 22.7 months (range: 1.8–180.1 months). The median OS for the entire cohort was 30.1 months (95% CI: 21.5–110.7 months), with a one-year OS rate of 78.3% (95% CI: 66.7–86.8) and a two-year OS rate of 57.4% (95% CI: 43.9–68.8). The median PFS was 8.9 months (95% CI: 6.3–17.7 months), with a 1-year PFS rate of 44.0% (95% CI: 32.0–55.3) and a 2-year PFS rate of 32.8% (95% CI: 21.9–44.2) (Figure 1A,B).

**Figure 2:**
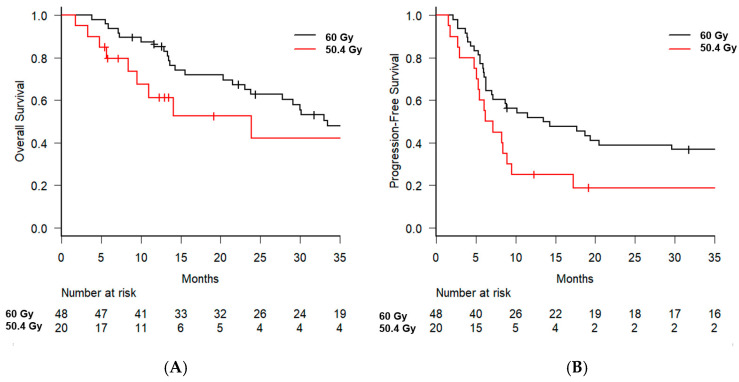
The Kaplan–Meier curves for overall survival (**A**) and progression-free survival (**B**), stratified by radiation dose.

**Figure 3:**
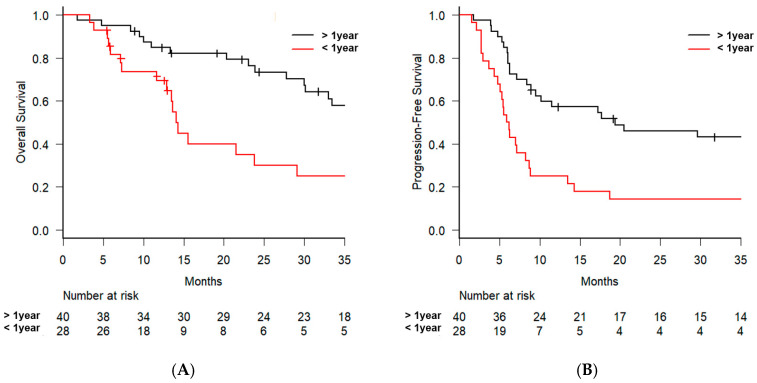
The Kaplan–Meier curves for overall survival (**A**) and progression-free survival (**B**) stratified by recurrence interval. Patients were stratified according to the interval from surgery to recurrence as follows: >1 year (black line) and <1 year (red line).

Subgroup analysis was conducted to identify the prognostic factors for OS. In the univariate analysis, significant factors included sex (male vs. female, hazard ratio [HR] = 0.283, 95% CI: 0.087–0.926, *p* = 0.037) and the interval to recurrence (≤1 year vs. >1 year, HR = 2.501, 95% CI: 1.284–4.869, *p* = 0.007) (Table 2). The 2-year OS rate was 62.7% (95% CI: 47.0–75.0%) in the 60 Gy arm, which was higher than the 42.0% (95% CI: 16.0–66.4%) observed in the 50.4 Gy arm; however, the difference was not statistically significant (*p* = 0.285) (Figure 2A). Additionally, the 2-year OS rate for patients with recurrence occurring >1 year after surgery was 73.4% (95% CI: 56.1–84.8%), whereas for those with recurrence within 1 year, the rate was significantly lower at 29.9% (95% CI: 12.6–49.6%, *p* = 0.0054) (Figure 3A). In the multivariate analysis, the interval to recurrence (≤1 year vs. >1 year) remained significant, with an HR of 2.307 (95% CI: 1.118–4.759, *p* = 0.024), along with radiotherapy dose (60 Gy vs. 50.4 Gy, HR = 2.414, 95% CI: 1.039–5.610, *p* = 0.040). No significant differences in patient characteristics were observed between the 60 Gy and 50.4 Gy arms (Supplementary Table S1).

For PFS, univariate analysis identified the interval to recurrence (≤1 year vs. >1 year) as a significant factor (HR = 2.494, 95% CI: 1.421–4.377, *p* = 0.001) (Table 3). In the Kaplan–Meier curve analysis, the 2-year PFS showed a trend toward better outcomes for patients in the 60 Gy arm; however, the difference was not statistically significant (38.9% vs. 18.7%, *p* = 0.064) (Figure 2B). For patients with recurrence occurring >1 year after surgery, the 2-year PFS rate was 46.0% (95% CI: 29.8–60.8%). In contrast, for patients with recurrence within 1 year, the 2-year PFS rate was significantly lower at 14.3% (95% CI: 4.5–29.5%, *p* = 0.001) (Figure 3B). In the multivariate analysis, the interval of recurrence (≤1 year vs. >1 year) remained significant, with an HR of 2.877 (95% CI: 1.576–5.250, *p* = 0.001), as along with radiotherapy dose (60 Gy vs. 50.4 Gy, HR = 2.547, 95% CI: 1.331–4.873, *p* = 0.005).

Treatment toxicity was retrospectively analyzed using the Common Terminology Criteria for Adverse Events (CTCAE) version 5.0 to assess patients’ adverse events. For acute adverse events, the most frequent Grade 3 or higher toxicity was leukopenia (Grade 3: 24 cases, Grade 4: 6 cases), followed by anemia (Grade 3: 10 cases) and thrombocytopenia (Grade 3: 5 cases, Grade 4: 3 cases). For late adverse events, three cases of Grade 3 toxicity were reported, including heart failure, pneumonia, and pleural effusion, with one case each. No Grade 4 or Grade 5 events were observed.

At the time of analysis, 43 patients showed disease progression after salvage CCRT. This included progression at the anastomotic site in three patients, regional lymph nodes in 26 patients, and distant metastases in 14 patients. Among these, three cases at the anastomotic site and 17 cases in the regional lymph nodes were within the salvage CCRT radiation field. The local control rate after salvage CCRT was 88.9% (95% CI: 78.1–94.6%) at 1 year and 71.9% (95% CI: 57.4–82.2%) at 2 years. Patients in the 60 Gy dose arm showed a trend toward better outcomes, but this was not statistically significant compared to the 50.4 Gy dose arm, with 1-year local control rates of 93.5% (95% CI: 81.0–97.8%) and 76.5% (95% CI: 48.8–90.4%), respectively (*p* = 0.0651). The most common site of distant metastasis was the lung, affecting five patients, followed by the liver and bone (three cases each). Additionally, pleural metastases were observed in two patients, and brain metastasis was identified in one patient. Following disease progression, 10 patients underwent curative local treatment (such as surgery or repeat CCRT), 19 received systemic therapy, 12 received best supportive care (BSC), and the treatment approach for 2 patients remains unknown. The median OS was 23.8 months for patients who underwent curative local treatment, 29.1 months for those who received systemic therapy, and 7.2 months for those managed with BSC.

## 4. Discussion

Loco-regional recurrence (LRR) of esophageal cancer following esophagectomy remains a significant therapeutic option, with limited options for curative intent. This study demonstrates that CCRT is a viable salvage treatment option with favorable survival outcomes.

Our OS and PFS results were consistent with those reported in recent studies, demonstrating comparable outcomes. Cho et al. reported 147 patients treated with salvage radiotherapy (RT) for LRR after surgery, achieving a median OS of 18.8 months and a PFS of 8.4 months [18]. While CCRT did not significantly improve OS compared to RT alone, it showed a trend toward better PFS but was associated with increased grade 3 or higher toxicities. Similarly, Torii et al. observed 2-year OS and PFS rates of 50.3% and 23.5%, respectively, in a cohort receiving salvage RT [19]. Ito et al. demonstrated that CCRT, with 60 Gy in 30 fractions, provided a median PFS of 8.2 months and OS of 23.1 months for postoperative LRR [20]. Mummudi et al. performed a systematic review and meta-analysis of 30 studies involving 1553 patients who received salvage radiotherapy with or without concomitant chemotherapy [21]. The pooled analysis revealed 1-, 2-, and 3-year OS probabilities of 67.9%, 35.9%, and 30.6%, respectively.

Stereotactic radiotherapy is also a salvage treatment option, which is a means of realizing highly accurate radiotherapy using advanced technologies [22]. Stereotactic body radiation therapy (SBRT) has been proven to be an effective treatment option for lung and liver oligo-recurrences in recurrent esophageal carcinoma [23,24]. Seyedin et al. evaluated the feasibility of SBRT as a salvage therapy for LRR of esophageal cancer after initial curative treatment [25]. Among the nine patients treated with a median SBRT dose of 27.5 Gy, SBRT showed minimal toxicity (no grade 3 or higher events) and durable treated tumor control, with a median PFS of 5.0 months and OS of 12.9 months.

Surgical re-resection is rarely feasible due to the technical difficulties and high complication rates associated with revision surgery in the thoracic cavity. Schipper et al. reported that re-resection of locally recurrent esophageal carcinoma was associated with significant morbidity, including 7% operative mortality and a 59% complication rate [26]. However, long-term survival is achievable with 2-, 3-, and 5-year survival rates of 62%, 44%, and 35%, respectively.

Our study revealed no significant differences between the chemotherapy regimens. However, the optimal concurrent chemotherapy regimen for esophageal cancer remains unknown. A phase 3 trial compared capecitabine or capecitabine plus oxaliplatin (XELOX) with fluorouracil plus cisplatin (PF) in definitive CCRT for inoperable locally advanced esophageal squamous cell carcinoma [27]. The 2-year OS rates were 75%, 66.7%, and 70.9% for capecitabine, XELOX, and PF, respectively, with no significant differences among them. Capecitabine was associated with fewer adverse events greater than Grade 3 (28.8%) than XELOX (36.5%) or PF (45.7%). Another phase III trial compared paclitaxel plus fluorouracil versus PF in definitive CCRT for locally advanced esophageal squamous cell carcinoma (ESCC) [28]. The 3-year OS rates were similar (55.4% vs. 51.8%, *p* = 0.448), with no significant difference in PFS. A phase II prospective study compared the efficacy and toxicity of docetaxel and cisplatin (DP) and FP regimens with CCRT in patients with ESCC [29]. The 5-year OS rates were 62.9% and 52.7% for the PF and DP groups, respectively, with no significant differences. Similarly, the 5-year PFS rates were 43.9% and 40.0% for PF and DP, respectively.

Our results indicate a preferred outcome in the high-dose radiation arm, which may have been influenced by a patient selection bias. There is no consensus on the optimal radiation dose for salvage CCRT. Regarding initial treatment, the INT 0123 phase III trial compared high-dose (64.8 Gy) and standard-dose (50.4 Gy) radiation therapy combined with 5-FU and cisplatin for esophageal cancer, and found no significant difference in survival or local/regional control between the two doses [30]. You et al. conducted a randomized study comparing high-dose (59.4 Gy) and standard-dose (50.4 Gy) IMRT in patients with inoperable thoracic esophageal squamous cell carcinoma and found no significant difference in the median OS (28.1 vs. 26.0 months, *p* = 0.54) [31]. Further studies are required to determine the optimal dose and fractionation schedule for salvage CCRT.

This study was limited by its retrospective design and the heterogeneity in treatment regimens, patient selection, and follow-up protocols. Chemotherapy regimens were not standardized and were selected at the discretion of the treating physician, leading to potential selection bias. Since 75% of patients received the NDP + S-1 regimen, meaningful comparisons across different chemotherapy groups were limited. Additionally, variations in administration and dosing schedules may have influenced treatment outcomes. Additionally, biopsy confirmation of recurrence is not universally performed, which may introduce bias into the assessment of treatment outcomes. Slight or mild adverse events were hardly assessed retrospectively, which is a limitation. Future prospective trials are required to validate the efficacy of salvage CCRT, evaluate the role of advanced radiotherapy techniques, and explore the integration of systemic therapies, including immunotherapy, in this setting.

## 5. Conclusions

Salvage CCRT offers a promising treatment option for patients with LRR of esophageal cancer following esophagectomy, providing favorable local control and survival outcomes. In this study, the time interval from initial treatment to recurrence and high-dose radiotherapy were identified as prognostic factors. While treatment intensification strategies based on recurrence timing as a prognostic indicator may be considered, further research is needed to validate this approach. Additionally, although higher radiation doses were associated with better outcomes, this finding is based on a retrospective analysis, and prospective studies are required for further validation.

## Figures and Tables

**Table 1:** Baseline characteristics and treatment details of 68 patients in this study.

Variables		Number (Percentage)
Age: median [range]		68 [41–83]
Sex		
	Male	56 (82%)
	Female	12 (18%)
ECOG-PS		
	0	56 (82%)
	1	12 (18%)
Initial anatomical segments		
	Cervical	6 (9%)
	Thoracic	47 (69%)
	Abdominal	2 (3%)
	Unknown	3 (4%)
Recurrence pattern		
	Anastomotic	13 (19%)
	Cervical lymph nodes	15 (22%)
	Thoracic lymph nodes	27 (40%)
	Abdominal lymph nodes	13 (19%)
Concurrent chemotherapy		
	CDDP + 5-FU	7 (10%)
	NDP + 5-FU	8 (12%)
	NDP + S-1	51 (75%)
	NDP	2 (3%)
Dose and fractionation		
	50.4 Gy in 28 fractions	28 (41%)
	60 Gy in 30 fractions	40 (59%)
Interval from surgery to recurrence		12.8 months [3.2–227.3]

CDDP: cisplatin, NDP: nedaplatin.

**Table 2:** Analysis of overall survival using univariate and multivariate Cox proportional hazards models.

Covariables		Univariate		Multivariate	
		Hazard Ratio [95% CI]	*p* Value	Hazard Ratio [95% CI]	*p* Value
Age	≤70 vs. >70 years old	0.646 [0.311–1.344]	0.242	0.633 [0.297–1.350]	0.236
Sex	Male vs. female	0.283 [0.087–0.926]	0.037	0.309 [0.088–1.085]	0.067
ECOG PS	0 vs. 1	0.928 [0.360–2.397]	0.878	1.312 [0.485–3.549]	0.593
Concurrent chemotherapy	NDP + S-1 vs. Others	1.395 [0.689–2.824]	0.355	1.309 [0.617–2.779]	0.483
Radiotherapy dose	60 Gy vs. 50.4 Gy	1.515 [0.704–3.261]	0.289	2.414 [1.039–5.610]	0.040
Recurrence pattern	Anastomotic vs. others	0.817 [0.337–1.981]	0.654	1.313 [0.516–3.337]	0.568
Interval of recurrence	>1 vs. ≤1 year	2.501 [1.284–4.869]	0.007	2.307 [1.118–4.759]	0.024

NDP: nedaplatin.

**Table 3:** Analysis of progression-free survival using univariate and multivariate Cox proportional hazards models.

Covariables		Univariate		Multivariate	
		Hazard Ratio [95% CI]	*p* Value	Hazard Ratio [95% CI]	*p* Value
Age	≤70 vs. >70 years old	0.751 [0.413–1.364]	0.347	0.612 [0.324–1.154]	0.129
Sex	Male vs. female	0.571 [0.267–1.221]	0.148	0.601 [0.266–1.355]	0.220
ECOG PS	0 vs. 1	1.371 [0.664–2.834]	0.394	1.851 [0.802–4.273]	0.149
Concurrent chemotherapy	NDP + S-1 vs. others	1.086 [0.573–2.058]	0.800	1.056 [0.544–2.051]	0.872
Radiotherapy dose	60 Gy vs. 50.4 Gy	1.756 [0.958–3.218]	0.068	2.547 [1.331–4.873]	0.005
Recurrence pattern	Anastomotic vs. others	0.983 [0.470–2.053]	0.963	1.233 [0.529–2.874]	0.627
Interval of recurrence	>1 vs. ≤1 year	2.494 [1.421–4.377]	0.001	2.877 [1.576–5.250]	0.001

NDP: nedaplatin.

## Data Availability

The datasets used for analysis during the current study are available from the corresponding author on reasonable request.

## Supplementary Materials

The following supporting information can be downloaded at: https://www.mdpi.com/article/10.3390/jcm14051540/s1, Supplementary Table S1: Baseline Characteristics of Patients Stratified by Radiation Dose (60 Gy vs. 50.4 Gy).

## References

[1] Zhang Y., Zhang Y., Peng L., Zhang L. (2022). Research Progress on the Predicting Factors and Coping Strategies for Postoperative Recurrence of Esophageal Cancer. Cells.

[2] Sudarshan M. (2019). Locoregional and oligometastatic recurrence of esophageal cancer-what are the management strategies?. J. Thorac. Dis..

[3] Su X.D., Zhang D.K., Zhang X., Lin P., Long H., Rong T.H. (2014). Prognostic factors in patients with recurrence after complete resection of esophageal squamous cell carcinoma. J. Thorac. Dis..

[4] Parry K., Visser E., van Rossum P.S., Mohammad N.H., Ruurda J.P., van Hillegersberg R. (2015). Prognosis and Treatment After Diagnosis of Recurrent Esophageal Carcinoma Following Esophagectomy with Curative Intent. Ann. Surg. Oncol..

[5] Sugawara K., Oka D., Hara H., Yoshii T., Ushijima H., Kudo S., Fukuda T. (2023). Survival outcomes of esophageal cancer patients with recurrence after curative treatments. BMC Cancer.

[6] Mitamura A., Tsujinaka S., Nakano T., Sawada K., Shibata C. (2024). Treatment Strategies for Locoregional Recurrence in Esophageal Squamous-Cell Carcinoma: An Updated Review. Cancers.

[7] Epistola R.J., Chao J. (2020). Systemic therapy for advanced gastroesophageal cancers: Progress and pitfalls. Transl. Gastroenterol. Hepatol..

[8] Hirano H., Kato K. (2019). Systemic treatment of advanced esophageal squamous cell carcinoma: Chemotherapy, molecular-targeting therapy and immunotherapy. Jpn. J. Clin. Oncol..

[9] Sun J.M., Shen L., Shah M.A., Enzinger P., Adenis A., Doi T., Kojima T., Metges J.P., Li Z., Kim S.B. (2021). Pembrolizumab plus chemotherapy versus chemotherapy alone for first-line treatment of advanced oesophageal cancer (KEYNOTE-590): A randomised, placebo-controlled, phase 3 study. Lancet.

[10] Doki Y., Ajani J.A., Kato K., Xu J., Wyrwicz L., Motoyama S., Ogata T., Kawakami H., Hsu C.H., Adenis A. (2022). Nivolumab Combination Therapy in Advanced Esophageal Squamous-Cell Carcinoma. N. Engl. J. Med..

[11] Shimada M., Itamochi H., Kigawa J. (2013). Nedaplatin: A cisplatin derivative in cancer chemotherapy. Cancer Manag. Res..

[12] Yang X., Ren H., Li Z., Zhang L., Shao Y., Li H., Sun Y., Zhang X., Wang Z., Fu J. (2022). A phase III randomized, controlled trial of nedaplatin versus cisplatin concurrent chemoradiotherapy in patients with cervical cancer. ESMO Open.

[13] Lu S., Chen Z., Hu C., Zhang J., Chen Y., Song Y., Zhao Q., Fan Y., Wu G., Ma Z. (2018). Nedaplatin Plus Docetaxel Versus Cisplatin Plus Docetaxel as First-Line Chemotherapy for Advanced Squamous Cell Carcinoma of the Lung—A Multicenter, Open-label, Randomized, Phase III Trial. J. Thorac. Oncol..

[14] Kubota T. (2008). The role of S-1 in the treatment of gastric cancer. Br. J. Cancer.

[15] Kang Y.K., Kim H.D., Yook J.H., Park Y.K., Lee J.S., Kim Y.W., Kim J.Y., Ryu M.H., Rha S.Y., Chung I.J. (2024). Neoadjuvant Docetaxel, Oxaliplatin, and S-1 Plus Surgery and Adjuvant S-1 for Resectable Advanced Gastric Cancer: Updated Overall Survival Outcomes From Phase III PRODIGY. J. Clin. Oncol..

[16] Endo S., Terazawa T., Goto M., Tanaka R., Kato T., Fujitani K., Kawakami H., Sakai D., Kurokawa Y., Tsujinaka T. (2022). Neoadjuvant docetaxel, oxaliplatin and S-1 therapy for the patients with large type 3 or type 4 gastric cancer (OGSG1902): Protocol of a multi-center, phase II study. BMC Cancer.

[17] Katano A., Yamashita H., Nakagawa K. (2019). Successful definitive concurrent chemoradiotherapy in a patient with esophageal cancer and Child-Pugh B cirrhosis of the liver. J. Cancer Res. Ther..

[18] Cho W.K., Noh J.M., Oh D., Ahn Y.C., Sun J.M., Kim H.K., Shim Y.M. (2024). Salvage Radiotherapy for Loco-Regional Recurrence of Esophageal Cancer Following Surgery. Cancer Res. Treat..

[19] Torii A., Tomita N., Takaoka T., Kondo T., Yamamoto S., Sugie C., Nagai A., Miyakawa A., Kuno M., Uchiyama K. (2024). Salvage radiotherapy for locoregional recurrence of esophageal cancer after surgery. Jpn. J. Clin. Oncol..

[20] Ito R., Nakamura Y., Sunakawa H., Fujiwara H., Hojo H., Nakamura N., Fujita T., Yano T., Daiko H., Akimoto T. (2022). Tumor response and survival outcomes of salvage concurrent chemoradiotherapy with three-dimensional conformal radiotherapy and 5-fluorouracil/platinum-based chemotherapy for postoperative locoregional recurrence of esophageal squamous cell carcinoma. Esophagus.

[21] Mummudi N., Jiwnani S., Niyogi D., Srinivasan S., Ghosh-Laskar S., Tibdewal A., Rane P., Karimundackal G., Pramesh C.S., Agarwal J.P. (2022). Salvage radiotherapy for postoperative locoregional failure in esophageal cancer: A systematic review and meta-analysis. Dis. Esophagus.

[22] Katano A., Minamitani M., Ohira S., Yamashita H. (2024). Recent Advances and Challenges in Stereotactic Body Radiotherapy. Technol. Cancer Res. Treat..

[23] Katano A., Yamashita H., Nakagawa K. (2017). Stereotactic body radiotherapy for oligo-recurrence in the liver in a patient with esophageal carcinoma: A case report. Mol. Clin. Oncol..

[24] Yamamoto T., Niibe Y., Matsumoto Y., Dekura Y., Oh R.J., Yamashita H., Kakuhara H., Aoki M., Jingu K. (2020). Stereotactic Body Radiotherapy for Pulmonary Oligometastases from Esophageal Cancer: Results and Prognostic Factors. Anticancer Res..

[25] Seyedin S.N., Gannon M.K., Plichta K.A., Abushahin L., Berg D.J., Arshava E.V., Parekh K.R., Keech J.C., Caster J.M., Welsh J.W. (2020). Safety and Efficacy of Stereotactic Body Radiation Therapy for Locoregional Recurrences After Prior Chemoradiation for Advanced Esophageal Carcinoma. Front. Oncol..

[26] Schipper P.H., Cassivi S.D., Deschamps C., Rice D.C., Nichols F.C., Allen M.S., Pairolero P.C. (2005). Locally recurrent esophageal carcinoma: When is re-resection indicated?. Ann. Thorac. Surg..

[27] Jia R., Shan T., Zheng A., Zhang Y., Lu P., Zhang G., Wang F., Xu Z., Zheng G., Tang D. (2024). Capecitabine or Capecitabine Plus Oxaliplatin Versus Fluorouracil Plus Cisplatin in Definitive Concurrent Chemoradiotherapy for Locally Advanced Esophageal Squamous Cell Carcinoma (CRTCOESC): A Multicenter, Randomized, Open-Label, Phase 3 Trial. J. Clin. Oncol..

[28] Chen Y., Ye J., Zhu Z., Zhao W., Zhou J., Wu C., Tang H., Fan M., Li L., Lin Q. (2019). Comparing Paclitaxel Plus Fluorouracil Versus Cisplatin Plus Fluorouracil in Chemoradiotherapy for Locally Advanced Esophageal Squamous Cell Cancer: A Randomized, Multicenter, Phase III Clinical Trial. J. Clin. Oncol..

[29] Jiang H., Makelike K., Chen B., Xi M., Li Q., Hu Y., Zhu Y. (2023). Definitive concurrent chemoradiotherapy with docetaxel plus cisplatin versus 5-fluorouracil plus cisplatin in patients with esophageal squamous cell carcinoma: Long-term follow-up results of a phase II randomized controlled trial. Radiat. Oncol..

[30] Minsky B.D., Pajak T.F., Ginsberg R.J., Pisansky T.M., Martenson J., Komaki R., Okawara G., Rosenthal S.A., Kelsen D.P. (2002). INT 0123 (Radiation Therapy Oncology Group 94-05) phase III trial of combined-modality therapy for esophageal cancer: High-dose versus standard-dose radiation therapy. J. Clin. Oncol..

[31] You J., Zhu S., Li J., Shen J., Zhao Y., Li X., Jia L., Li Q., Yang J., Wu Y. (2023). High-Dose Versus Standard-Dose Intensity-Modulated Radiotherapy With Concurrent Paclitaxel Plus Carboplatin for Patients With Thoracic Esophageal Squamous Cell Carcinoma: A Randomized, Multicenter, Open-Label, Phase 3 Superiority Trial. Int. J. Radiat. Oncol. Biol. Phys..

