# Supplementary material for: Salvage Chemoradiotherapy for Loco-Regional Recurrence of Esophageal Squamous Cell Carcinoma After Esophagectomy
Source: J Clin Med. 2025 Feb 25;14(5):1540. doi: 10.3390/jcm14051540 (PMC11899801; doi:10.3390/jcm14051540)
Supplement: Supplementary file 1 [file jcm-14-01540-s001.zip › jcm-3488868-supplementary.pdf]

Supplementary Data

Supplementary Table S1. Baseline Characteristics of Patients Stratified by Radiation Dose (60 Gy vs. 50.4 Gy)

Variables	Number (Percentage)		*p* value
	**60Gy/30fr arm**	**50.4Gy/28fr arm**	
Age: Median [Range]	67 [41-83]	71 [45-82]	0.342
Gender			
Male	40 (83%)	16 (80%)	0.737
Female	8 (17%)	4 (20%)	
ECOG-PS			
0	42 (88%)	14 (70%)	0.159
1	6 (13%)	6 (30%)	
Initial anatomical segments			
Cervical	3 (6%)	3 (15%)	0.277
Thoracic	42 (88%)	15 (75%)	
Abdominal	0 (0%)	2 (10%)	
Unknown	3 (6%)	0 (0%)	
Recurrence pattern			
Anastomotic	10 (21%)	3 (15%)	0.406
Cervical lymph nodes	11 (23%)	4 (20%)	
Thoracic lymph nodes	16 (33%)	11 (55%)	
Abdominal lymph nodes	11 (23%)	2 (10%)	
Concurrent chemotherapy			
CDDP+5-FU	5 (10%)	2 (10%)	0.586
NDP+5-FU	7 (15%)	1 (5%)	
NDP+S-1	34 (71%)	17 (85%)	
NDP	2 (4%)	0 (0%)	
Interval from surgery to recurrence	12.8 Months [3.8-55.1]	12.8 Months [3.2-227.3]	0.182

CDDP: Cisplatin, NDP: Nedaplatin.
